# Philosophy and neuroscience on consciousness – response to Felipe León and Dan Zahavi

**DOI:** 10.1007/s00701-023-05873-3

**Published:** 2023-11-10

**Authors:** Tobias A. Wagner-Altendorf

**Affiliations:** 1 Present Address: Department of Neurology, University of Lübeck, Ratzeburger Allee 160, 23538 Lübeck, Germany; 2 Munich School of Philosophy, Munich, Germany

Dear Editor,

León and Zahavi [3] have made a compelling case for the necessity of philosophy — and not only neuroscience — for investigating consciousness. In particular, they argue that any theory of consciousness cannot avoid philosophical enquiry and thus only can choose between good or bad philosophy. Also, the topics of self-consciousness and selfhood are highlighted as problems of consciousness *sui generis* next to the mind–body problem. I will try to elucidate a bit more the specific approaches to consciousness that philosophy and neuroscience take and thus elaborate why the philosophy and the neuroscience of consciousness are complimentary rather than mutually exclusive.

León and Zahavi [3] note that the philosophical question of the relation between mind and matter, as conceptualized, e.g., by the positions of dualism or physicalism, emergentism, eliminativism, or panpsychism, is “not a question that can be answered only by appeal to empirical evidence” (p. 834). I argue that this point should be made even stronger: It seems difficult to find a single philosophical position that is *ruled out* by empirical data,^1^ and even between the most opposite positions in the philosophy of mind cannot be decided by empirical means — take the positions of materialism and idealism as a striking example (see [7]).

Thus, the same set of empirical findings usually is compatible with various philosophical views on the relation between mind and matter. For example, the Integrated Information Theory of consciousness clearly fits with an identity theory (e.g., [6]) but also with panpsychism (e.g., [5]) — both of which are highly distinct philosophical positions. The Global Neuronal Workspace Theory, to name another prominent empirical consciousness theory holding that consciousness basically is “information broadcasting” in the parietal and prefrontal cortex (e.g., [4]), obviously is equally compatible with reductive physicalism or dualist (qualia) epiphenomenalism — again, two clearly differing philosophical conceptions. Given this (empirical) undecidability between philosophical positions, in the philosophy of mind, as Christof Koch has rightly noted, “highly polished arguments and counter-arguments are exchanged in a never-ending cycle that results in drawn-out sophisticated disagreements but no resolution” ([2], 73).^2^

What, then, are the consequences for the clinical neurologist or neurosurgeon confronted and dealing with disorders of consciousness and brain function? Again, in the same sense that a set of empirical findings is compatible with differing philosophical conceptions, a certain philosophical understanding does not predetermine a specific clinical approach. Various philosophical positions can potentially underline the *value* that consciousness has for our (patients’) lives and thus motivate clinicians to pursue a meaningful recovery from disorders of consciousness. I see no particular problem here for most of the positions in the philosophy of mind, including reductionist or emergentist, physicalist, idealist, or panpsychist approaches.

As far as I can see, only one philosophical position threatens to considerably downplay the intrinsic value of consciousness, and that is eliminative materialism or illusionism. If conscious experience per se — above functional disposition and behavioral output — does not exist, it cannot be of any value. The intuitively striking — but however behaviorally not detectable — difference between a (fully unconscious) patient in vegetative state and a (fully conscious) patient with locked-in syndrome would also be an illusion, given eliminative materialism. This, I claim, is an untenable consequence for the clinical neurologist or neurosurgeon dealing with disorders of consciousness, who must be realist about conscious experience.

## Footnotes

1 One might think that interactionist dualism is that single position, given its violation of the causal closure of the (micro-)physical. However, proponents of interactionism will strongly disagree here (see, e.g., Chalmers [1], 124ff., for a discussion of type-D dualism) — and, again, whether this disagreement is justified or not must be debated on conceptual, not empirical, grounds.

2 However, one should disagree with Koch’s subsequent statement that “the science of consciousness must [i.e., *can*] break out of these endless epicycles of arguments,” for the above stated reasons.
